# Priapism in the Newborn: Shall We Intervene?

**DOI:** 10.21699/jns.v6i1.389

**Published:** 2017-01-01

**Authors:** Rachida Laamiri, Nahla Kechiche, Mezri Maatouk, Besma Hagui, Walid Mnari, Mongi Mekki, Abdellatif Nouri

**Affiliations:** 1 Department of Pediatric Surgery, Fattouma Bourguiba University Hospital- Monastir, Tunisia; 2 Department of Radiology, Fattouma Bourguiba University Hospital-Monastir, Tunisia

**Keywords:** Neonatal, Priapism, Management

## Abstract

Idiopathic neonatal priapism is rarely published. We report the case of a newborn presenting with priapism on the first day of life and reviewed the published data on the management and the follow up of this condition.

## INTRODUCTION

Idiopathic neonatal priapism is an exceptional condition of a prolonged and persistent penile erection which is spontaneous and unassociated with catheterization or rectal stimulation. Due to its rarity, its etiology is unknown and there is no consensus regarding the management of newborn presenting idiopathic persisting erection. We present a case of idiopathic neonatal priapism.

## CASE REPORT

A (4-kg) newborn of a 34-year-old mother presented with persisting and painless erection on the first day of life. The prenatal screens were normal. He was born at term by an uncomplicated vaginal delivery. The physical exam clearly showed a healthy infant with persisting erection. The scrotum and the penis were not discolored and the two testes were palpable. The laboratory findings were normal. Doppler ultrasound of the penis showed normal arterial and venous flow. Masterly inactivity was pursued. The priapism decreased spontaneously and complete detumescence occurred on ninth day of life. The follow up from the first month until one year of age, revealed that the patient had normal erection during sleep.

## DISCUSSION

Priapism can be classified into two types: ischemic priapism (veno-occlusive) and non-ischemic priapism (arterial). In ischemic priapism, the erection is always painful; however, in the non-ischemic priapism, the erection is usually painless [1,2]. The etiology of neonatal priapism remains unclear. In the 18 cases reported in the literature, idiopathic priapism seemed to have been the most common etiology [3]. It results from a benign non-ischemic erection. The causes of secondary priapism are pharmacotherapy, neurological conditions, malignancy or trauma. The sickle cell disease is the most common cause of priapism in childhood but due to the fetal hemoglobin, priapism does not show up in newborn [3,4].

Clinically, it’s a prolonged painless penile erection with a quite variable duration ranging from two to twelve days [1,3]. Only one case of a newborn priapism complicated by a pyocavernositis after 20 days was reported. Most published cases showed the same clinical characteristics [5].

Untreated ischemic priapism causes fibrosis of the cavernous bodies that leads to erectile dysfunction and must therefore be treated promptly in all cases. In contrast, non-ischemic priapism, does not require urgent treatment, and may even be managed by observation [2].

All published cases reported positive outcomes with full functional recovery regardless of possible etiology, duration or treatment. This suggests that the management should not be exhaustive [1-4]. A penis Doppler ultrasound and a complete blood count are the first-line investigations; invasive explorations are unnecessary. Seventy five percent of cases reported in the literature were managed by close observation with spontaneous resolution as was the case of our patient without sequalae [3].

## Footnotes

**Source of Support:** Nil

**Conflict of Interest:** Nil
